# Successful treatment with spinal cord stimulation for pain due to eosinophilic granulomatosis with polyangiitis: a case report

**DOI:** 10.1186/s40981-023-00610-2

**Published:** 2023-04-06

**Authors:** Kumiko Tanabe, Yoko Sugiyama, Noritaka Yoshimura, Shinobu Yamaguchi

**Affiliations:** 1 Department of Anesthesiology and Pain Medicine, Gifu University Graduate School of Medicine, Gifu, 501-1194 Japan; 2 Anesthesiology and Pain Relief Center, Central Japan International Medical Center, Minokamo, Japan

**Keywords:** Chronic pain, Eosinophilic granulomatosis with polyangiitis, Spinal cord stimulation

## Abstract

**Background:**

Although most patients of eosinophilic granulomatosis with polyangiitis (EGPA) experience a reduction in pain within several weeks to months of the initiation of immunotherapies, some suffer from residual neuropathic symptoms for a long time.

**Case presentation:**

A 28-year-old woman diagnosed with EGPA visited. She had been treated with steroid pulse therapy, intravenous immunoglobulin, and mepolizumab (antiinterleukin-5 agent). Her symptoms other than peripheral neuropathy improved, but posterior lower thigh pain and weakness of the lower legs worsened. At the initial visit, she used crutches and complained of numb pain in both posterior lower thighs, especially the left one. She also presented with left foot drop and reported a decreased tactile sensation on the lateral sides of both lower thighs. We performed spinal cord stimulation (SCS) at the L1 level on both sides. Her pain remarkably decreased, her tactile sensation improved, her muscle strength increased, and she was able to walk without crutches.

**Conclusions:**

We herein report the first case of lower extremity pain being successfully treated with SCS in an EGPA patient who did not respond well to drug therapy. Because the cause of pain in EGPA is neuropathy induced by vasculitis, there is ample ability for SCS to improve this pain. When pain is neuropathic, whatever the cause, SCS may be worth trying, even for pain from disorders other than EGPA.

## Background

Eosinophilic granulomatosis with polyangiitis (EGPA) is an anti-neutrophil cytoplasmic antibody (ANCA)-associated vasculitis. EGPA is a rare disease, and its pathogenesis remains largely unknown [1, 2]. It is characterized systemic necrotizing vasculitis of small and medium-sized vessels induced by perivascular and extravascular granulomas and eosinophil infiltration [1, 2]. The clinical manifestations tend to segregate into eosinophilic disorder and vasculitis disorder, depending on the ANCA status [1, 2], with the former being more prevalent in the ANCA-negative population and the latter more prevalent in the ANCA-positive population [1, 2]. The manifestations are wide-ranging, both those induced by eosinophilic non-vasculitis, such as asthma, rhinosinusitis, peripheral and tissue eosinophilia, and cardiomyopathy, and that induced by vasculitis, such as purpura, glomerulonephritis, and mononeuritis multiplex [3]. The prevalence of peripheral neuropathy in EGPA patients is reportedly between 46 and 77% [4, 5]. Peripheral neuropathy in EGPA patients is characterized by polyneuropathy and mainly affects the lower extremities, with peroneal nerve involvement being the most frequent and severe [4]. The main manifestations of motor neuropathy are foot drop and muscle weakness, and sensory neuropathy is distributed mostly asymmetrically in the distal limbs [4].

Patients with EGPA have historically been treated by cyclophosphamide, glucocorticoids, and sometimes antirheumatic drugs, such as azathioprine, methotrexate, and mycophenolate mofetil, depending on the severity [3]. Mepolizumab, an antiinterleukin-5 agent, was the first FDA-approved agent, and other biologic agents have since been developed for the treatment of EGPA [3]. Although most patients experience a reduction in pain and improvement in their strength within several weeks to months of the initiation of immunotherapies, some suffer from residual neuropathic symptoms for a long time, even after EGPA remission [5].

Rituximab (anti-CD20 antigen on the surface of B cells) is reported to be effective for treating vasculitis manifestations, such as neuropathy [3], while mepolizumab and intravenous immunoglobulin are reported to be effective for treating neuropathy [3, 6]. Peripheral neuropathy does not affect the patient survival but significantly disrupts the quality of life because of muscle weakness and pain in the extremities. Multidisciplinary approaches, including rehabilitation and use of orthosis, are also recommended.

Although the mechanisms underlying the pain relief induced by spinal cord stimulation (SCS) are still not fully understood, this approach has been used in a variety of pathological states, including conditions involving peripheral neuropathic pain, such as complex regional pain syndrome, failed back surgery syndrome, diabetic neuropathy, ischemic pain, and postherpetic neuralgia [7]. Pain in EGPA patients is peripheral neuropathy induced by vasculitis of the small vessels that supply the terminal nerves [4]. Therefore, we speculated that SCS might be effective for pain of EGPA.

We herein report the first case of lower extremity pain being successfully treated with SCS in an EGPA patient who did not respond well to drug therapy.

## Case presentation

A 28-years-old woman diagnosed with EGPA was introduced to our hospital due to bilateral posterior thigh pain. She had asthma and atopic dermatitis. Six months ago, she had complained of left fingers numbness, pain from the left axilla to the sternum, and a fever. After 2 weeks, pain appeared in the right lower thigh and expanded to the entire lower extremities. Bilateral leg purpura and edema, a decreased grip strength with both hands, left tinnitus, and ear blockade sensation also appeared. Five months ago, she developed left drop foot, and a blood examination revealed the following values: eosinophile count, 4000/μL; C-reactive protein, 2.69 mg/dL; ANCA antibody, negative; and immunoglobulin E, 2.977 U/mL. She was therefore diagnosed with EGPA and treated with prednisolone.

After treatment, her symptoms other than peripheral neuropathy improved, but both the posterior lower thigh pain and weakness of the lower legs worsened. She received steroid pulse therapy and intravenous immunoglobulin, but her symptoms were not improved. Four months ago, mepolizumab (antiinterleukin-5 agent) was started, but her symptoms were unchanged.

After moving to a new house, she visited the Department of Internal Medicine in our hospital and was introduced to our department for pain treatment. At the first visit, she used crutches and complained of numb pain in both posterior lower thighs, especially the left. She presented with left foot drop on manual muscle testing with right ankle flexion 4 and extension 5 and lefts ankle flexion 0 and extension 4. She also had a decreased tactile sensation (moderate to severe) on the lateral sides of both lower thighs. She had been receiving subcutaneous mepolizumab 300 mg/month and oral prednisolone 10 mg/day, pregabalin 150 mg/day, duloxetine 40 mg/day, suvorexant 15 mg/day, vitamin B1/B6/B12, vonoprazan, alphacalcidol, ferrous citrate, trimethoprim/sulfamethoxazole, and alendronate, as well as inhaled budesonide. We added tramadol 100 mg/day and learned that left sciatic nerve block using local anesthetics with glucocorticoid at the popliteal fossa had provided pain relief for one week at the previous hospital. Pulsed radiofrequency (42 °C for 6 min) of the left sciatic nerve at the popliteal fossa was performed after several attempts at sciatic nerve blockade with local anesthetic. However, this was utterly ineffective. We then decided to attempt SCS and finally obtained good results.

**Fig. 1:**
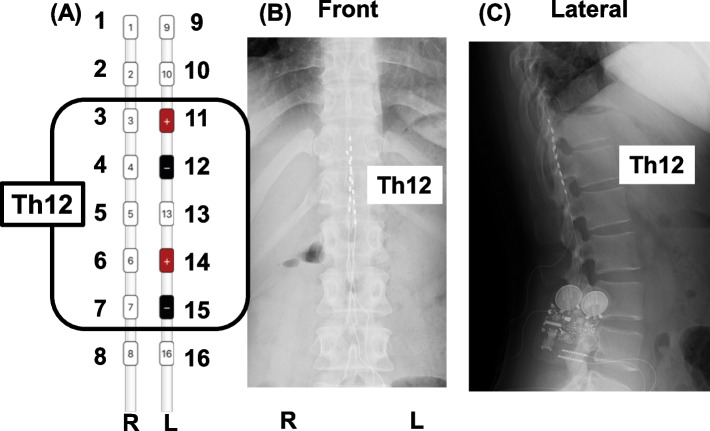
The two percutaneous trial lead positions. **A** An enlarged schematic view of the stimulation site. **B** Plain X-ray front view. **C** Plain X-ray lateral view. Electrodes number 11, 12, 14, and 15, which correspond to the left-side Th12 level, were used for stimulation. R, right side; L, left side

The details of SCS are described here. Two percutaneous eight-contact trial leads, Octorode (Abbott, Plano, TX, USA), were inserted spanning the Th11-Th12 epidural space (Fig. 1). An external pulse generator was programmed using passive burst stimulation according to standard programming techniques. Only electrodes numbers 11, 12, 14, and 15, which correspond to the left-side Th12 level, were used for stimulation according to the pain region after determining the appropriate stimulation position using tonic stimulation. BustDR™ (Abbot) fixes stimulation at 5 pulses per burst, with a 500-Hz intraburst frequency, 40-Hz interburst frequency, and 1000-μsec pulse width, continuously delivered. The patient was treated with a stimulus intensity of 0.35 mA and maximum intensity of 0.4 mA, adjusted to the pain intensity.

**Fig. 2:**
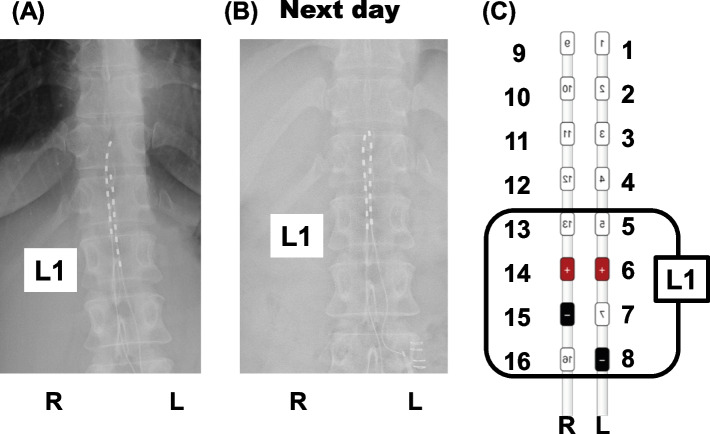
The two permanent lead positions. **A** Plain X-ray front view on the operation day. **B** Plain X-ray front view day after the operation. **C** An enlarged schematic view of the stimulation site. Finally, electrodes number 6, 8, 14, and 15, which correspond to the L1 level on both sides, were used for stimulation. R, right side; L, left side

Her leg pain improved over the 10 days of stimulation, but the pain gradually increased again after the leads were removed. Three months after this successful trial, the patient had two eight-contact leads, Proclaim (Abbott), permanently implanted into the epidural space (Fig. 2). The cranial end of the right lead was located over the middle of the Th11 vertebral body and that of the left lead was located at the upper edge of the Th12 vertebral body. Electrodes numbers 6 and 8, which correspond to the left-side L1 level, were used for stimulation according to the pain region. We examined X-ray findings at the day after implantation and found that the cranial end of the right lead had moved to the upper edge of the Th12 vertebral body. Immediately after implantation, the patient was stimulated at an intensity of 0.2 mA (maximum intensity 0.45 mA) and adjusted to the pain intensity. After 10 days, electrodes numbers 14 and 15, which correspond to the right-side L1 level, were added for stimulation.

Her pain remarkably decreased, and the intensity of stimulation was able to be decreased to a stimulus intensity of 0.15 mA (maximum intensity 0.25 mA) by 6 months later. After 1 year, her legs pain had almost completely disappeared, and she showed improved tactile sensation, increased of muscle strength, and the ability to walk without crutches. Neurologists were able to stop administrating medications for her pain (pregabalin, duloxetine, and tramadol) and decrease the prednisolone dose. The most recent stimulus condition has since been continued, and we are planning reconsider it depending on her condition going forward.

The patient provided her permission for the presentation of this report.

## Discussion

EGPA is characterized histologically by eosinophilic infiltration and vasculitis [3]. The positive rate of ANCA in EGPA is lower than that in other ANCA-associated vasculitis conditions [5]. Our patient was ANCA-negative. Compared with ANCA-positive patients, the vessels in ANCA-negative EGPA patients are more frequently filled with eosinophils, despite the structures in the vascular wall being preserved, and eosinophils are more frequently observed in the extravascular space of the endoneurium [5]. Although necrotizing vasculitis is more frequently observed in ANCA-positive EGPA patients than in ANCA-negative patients, the extent of nerve fiber degeneration is similar in both groups of patients [5]. Thus, tissue damage induced by eosinophiles might be involved in the mechanism underlying neuropathy in EGPA [5].

Two hypothetical mechanisms are suggested as follows: (1) eosinophils interrupt blood circulation by clogging the small vessels that supply the terminal nerves and (2) eosinophils induce toxicity by releasing proteins within the eosinophilic-specific granules into the extracellular milieu [4, 5]. Thus, EGPA frequently causes mononeuritis multiplex, and the symptoms are severe in some cases and require aggressive immunosuppressive therapies [8]. Although our patient had been treated with prednisolone, steroid pulse therapy, intravenous immunoglobulin, mepolizumab, and received pain medications (pregabalin, duloxetine, and tramadol) for a long time, her leg pain was still severe. We therefore judged that the improvement of her symptoms after SCS implantation was due to the effects of SCS and not the natural healing process.

SCS is an established therapy for chronic neuropathic pain, including neuropathy associated with diabetic mellites, human immunodeficiency virus, and chemotherapy [9]. However, there have been no reports of the utility of SCS treatment for neuropathic pain due to EGPA. As mentioned above, because the cause of pain in EGPA is neuropathy induced by vasculitis, there is every possibility that SCS might improve this pain. When the pain is neuropathic, whatever the cause, SCS may be worth trying, even for pain from disorders other than EGPA. Although the exact effects of SCS on the microcirculatory system are unknown, SCS is known to heal ischemic skin ulcer in scleroderma patients [10] and salvages ischemic limbs in patients with peripheral vascular disease [11]. Thus, it has been speculated that SCS improves microcirculation. In our case, SCS may have improved the microcirculation supplying the terminal nerves.

Several paradigms for SCS have been suggested thus far (tonic, burst, and high frequency), with the main differences being the presence of paresthesia by the treatment [9]. We selected high-frequency burst stimulation in the present case. This type of stimulation is intended to mimic naturally occurring neuronal firing within the central nervous system [12] and is characteristically paresthesia-free [9]. However, which types of stimulation are most suitable for which types of pain remains unclear. In addition, our patient experienced dramatic improvement in both pain and movement. Her movement disorder improved partially because of the pain improvement. Furthermore, some researchers report that movement disorders, such as gait and posture, improve with SCS alone [9]. However, the indications of SCS for movement disorders have not yet been established.

This is the first report of successful pain treatment of EGPA by SCS. SCS should be considered a potential treatment option for intractable neuropathic pain, regardless of the cause.

## Data Availability

Data sharing is not applicable to this article as no datasets were generated or analyzed during the current study.

## Abbreviations

EGPA: Eosinophilic granulomatosis with polyangiitis
ANCA: Anti-neutrophil cytoplasmic antibody
SCS: Spinal cord stimulation
